# Decoration of green synthesized S, N-GQDs and CoFe_2_O_4_ on halloysite nanoclay as natural substrate for electrochemical hydrogen storage application

**DOI:** 10.1038/s41598-022-12321-2

**Published:** 2022-05-16

**Authors:** Maryam Ghiyasiyan-Arani, Masoud Salavati-Niasari

**Affiliations:** Institute of Nano Science and Nano Technology, University of Kashan, P. O. Box. 87317-51167, Kashan, Islamic Republic of Iran

**Keywords:** Energy, Synthesis and processing, Hydrogen storage materials, Composites, Natural product synthesis

## Abstract

Halloysite nanotubes (HNTs) with high active sites are used as natural layered mineral supports. Sulfur- and nitrogen-co doped graphene quantum dots (S, N-GQDs) as conductive additive and CoFe_2_O_4_ as the electrocatalyst was decorated on a HNT support to design an effective and environmentally friendly active material. Herein, an eco-friendly CoFe_2_O_4_/S, N-GQDs/HNTs nanocomposite is fabricated via a green hydrothermal method to equip developed hydrogen storage sites and to allow for quick charge transportation for hydrogen storage utilization. The hydrogen storage capacity of pure HNTs was 300 mAhg^−1^ at a current density of 1 mA after 20 cycles, while that of S, N-GQD-coated HNTs (S, N-GQDs/HNTs) was 466 mAhg^−1^ under identical conditions. It was also conceivable to increase the hydrogen sorption ability through the spillover procedure by interlinking CoFe_2_O_4_ in the halloysite nanoclay. The hydrogen storage capacity of the CoFe_2_O_4_/HNTs was 450 mAhg^−1^, while that of the representative designed nanocomposites of CoFe_2_O_4_/S, N-GQDs/HNTs was 600 mAhg^−1^. The halloysite nano clay and treated halloysite show potential as electrode materials for electrochemical energy storage in alkaline media; in particular, ternary CoFe_2_O_4_/S, N-GQD/HNT nanocomposites prove developed hydrogen sorption performance in terms of presence of conductive additive, physisorption, and spillover mechanisms.

## Introduction

Clay minerals have been widely exploited for expanding functional nano compounds, owing to the diversity of these silicates to assemble various types of acting species at nano size^1,2^. Accordingly, different materials, such as nanoparticles, polymers or biological components, can be assembled to create highly diverse nanoconstructions of interest as clay nanocomposites for applications in energy storage, catalyst reactions and sensors, among other demands^3,4^.

Several reports have been considered for applying clay minerals for energy storage applications in different energy storage devices, such as membranes, electrolytes and electrodes. Halloysite nanotubes (HNTs) are a tubular clay with structural details of 50 nm in diameter, 600–900 nm in length and 15 nm for the inner lumen^5,6^. Halloysite is a distinctive material that is constructed by rolling kaolin sheets into multiwalled mesoporous nanotubes. The surface of halloysite is created by alumina (lumen surfaces) and silica (other surfaces)^7,8^. Its outstanding features include hollow construction, high porosity, tunable surface characteristics, low cost, versatile surface functionalization and easy preparation^9–13^. Surface modification of halloysite nanotubes can be conduct in outer surface or inner lumen. The type of surface modification strategy to be chosen can depend on the type of application required. These modification agents include polymer, nanoparticles, surfactants, Organosilane coupling, Acid etching and Compounds of biological origin^14^.

Energy and power generations are the basic topics to manage the suitability of industrial society and human lifetime. All actions need energy, comprising of making products, chilling or warming the buildings, driving, and lighting motor systems^15^. Currently, we rely on energy originations that are nonrenewable to meet most energy requirements. These nonrenewable originations are quickly discharging due to their vast operation^16^. Additionally, these sources are an essential basis for changing the weather because these fossil combustions are released Carbon dioxide (CO_2_), which is absolutely destructive to the surroundings^17^. So, hydrogen energy can be the most challenging source for energy devices in the future^18^. Hydrogen is identified as a nontoxic and low-priced power origin for static and transferable avails. Hydrogen supplies reproducible energy after ignition^19^. Scientists have drawn consideration to hydrogen as an encouraging future fuel because of its substantial features. These features are, containing plenty in the world, an undefiled energy source, and the lightest fuel^20,21^.

Electrode material properties are crucial for the energy storage performance of storage devices. It has been well appointed that nanostructured electrode materials can enhance the capacity and cycle stability of storage devices. Mass transport and electrode kinetics can be elevated by decreasing the charge transportation path and ion diffusion distance. In addition, the cooperation of several mechanisms and storage processes can promote the potential of electrode materials for ion adsorption. In layered materials, especially clay minerals with unique structures, a spillover mechanism can occur by decorating electrocatalyst materials on their surfaces. Adsorbed H_2_ molecules on the decorated electrocatalyst migrate to the surface of clay minerals, and hydrogen can be transported on a surface to another surface. Additionally, designing carbon bridges between the dissociation source (electrocatalyst) and receptor (clay) leads to diffusion. Graphene quantum dots (GQDs), as a functional carbon source with distinctive physicochemical properties, play ideal roles in the energy storage process^22,23^. The presence of GQDs in the texture of electrodes has been reported in previous studies for studying electrochemical energy storage systems such as Li-ion batteries, Li–S batteries, and supercapacitors^24–28^. Yushan Liu groups reposted the preparation of HNTs/RGO nanocomposites which provided superior performances as electrode material in supercapacitors^29^. Nano assembly of N-doped graphene quantum dots anchored Fe_3_O_4_/halloysite nanotubes designed by Akhilesh BabuGanganboina et.al. for high performance supercapacitor^30^.

According to the mentioned literature, the HNT nanoclay, CoFe_2_O_4_ and S, N-GQDs were selected as support, electrocatalyst and conductive receptor components, respectively. Hence, ternary nanocomposites were designed by decorating CoFe_2_O_4_ and S, N-GQDs on halloysite nanotubes. CoFe_2_O_4_ as a transition metal oxide in the category of spinel materials has been widely utilized in electrochemical energy storage systems^31,32^. Electrochemical energy storage devices undergo redox reactions with high power, which store electrical charges at the surface and in the structure pores^33^. The achieved ternary CoFe_2_O_4_/S, N-GQD/HNT nanocomposites were prepared through a green hydrothermal method with synergistic spillover mechanisms, redox processes and physical adsorption as potential hydrogen storage materials. Decoration of the S, N-GQD layer as a conductive receptor and CoFe_2_O_4_ nanoparticles as an electrocatalyst on halloysite nanotubes was investigated using structural analysis of XRD, FE-SEM, TEM, FT-IR and EDS. The electrochemical abilities of the resulting composites were compared to reach ideal electrode materials in terms of function, mechanism and performance.

## Results

### Structure characterization

**Figure 1:**
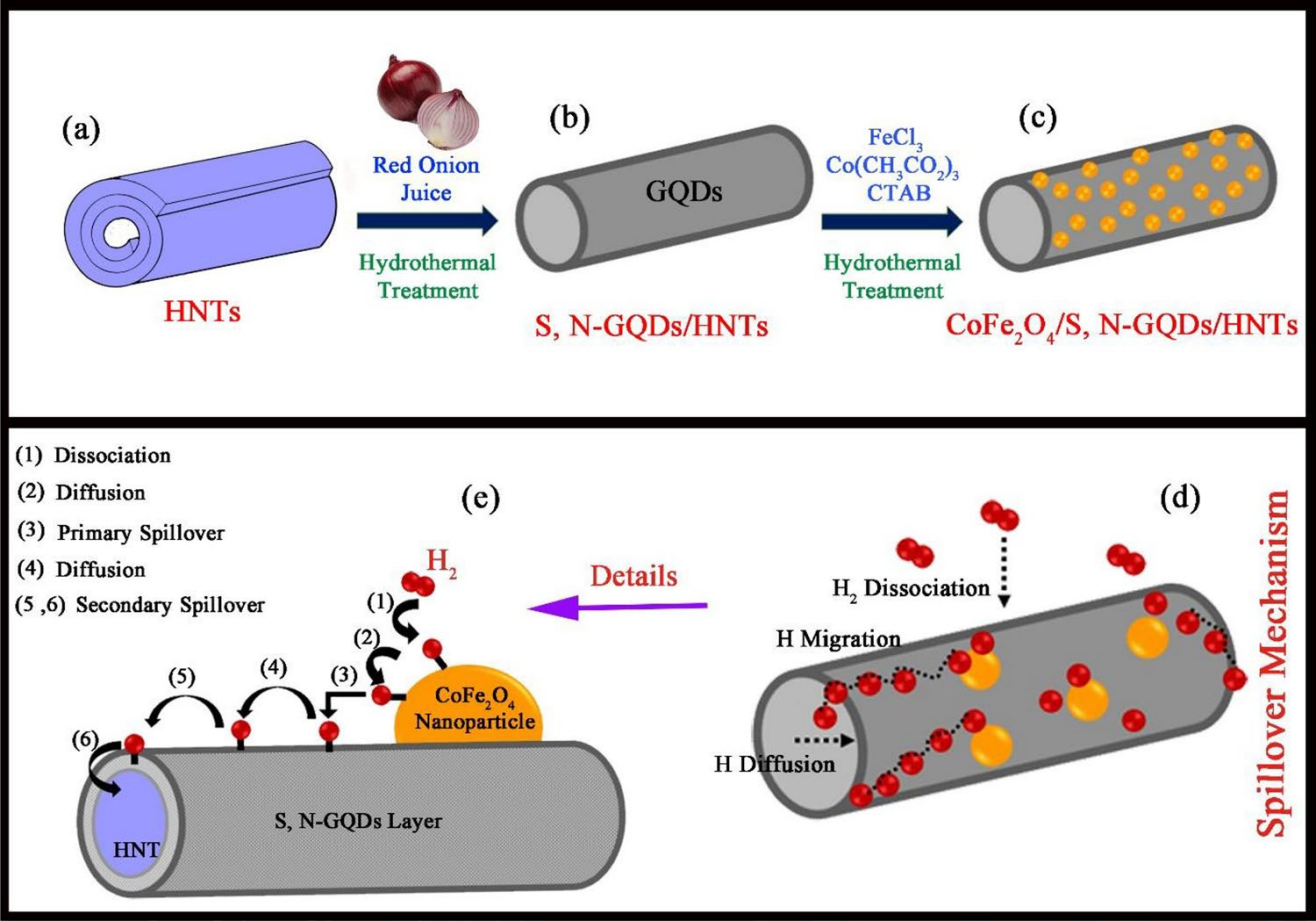
(**a**–**c**) Decoration of S, N-GQDs and CoFe_2_O_4_ nanoparticles on the HNT substrate and (**d**, **e**) Spillover mechanism of CoFe_2_O_4_/S, N-GQDs/HNTs for hydrogen storage.

**Figure 2:**
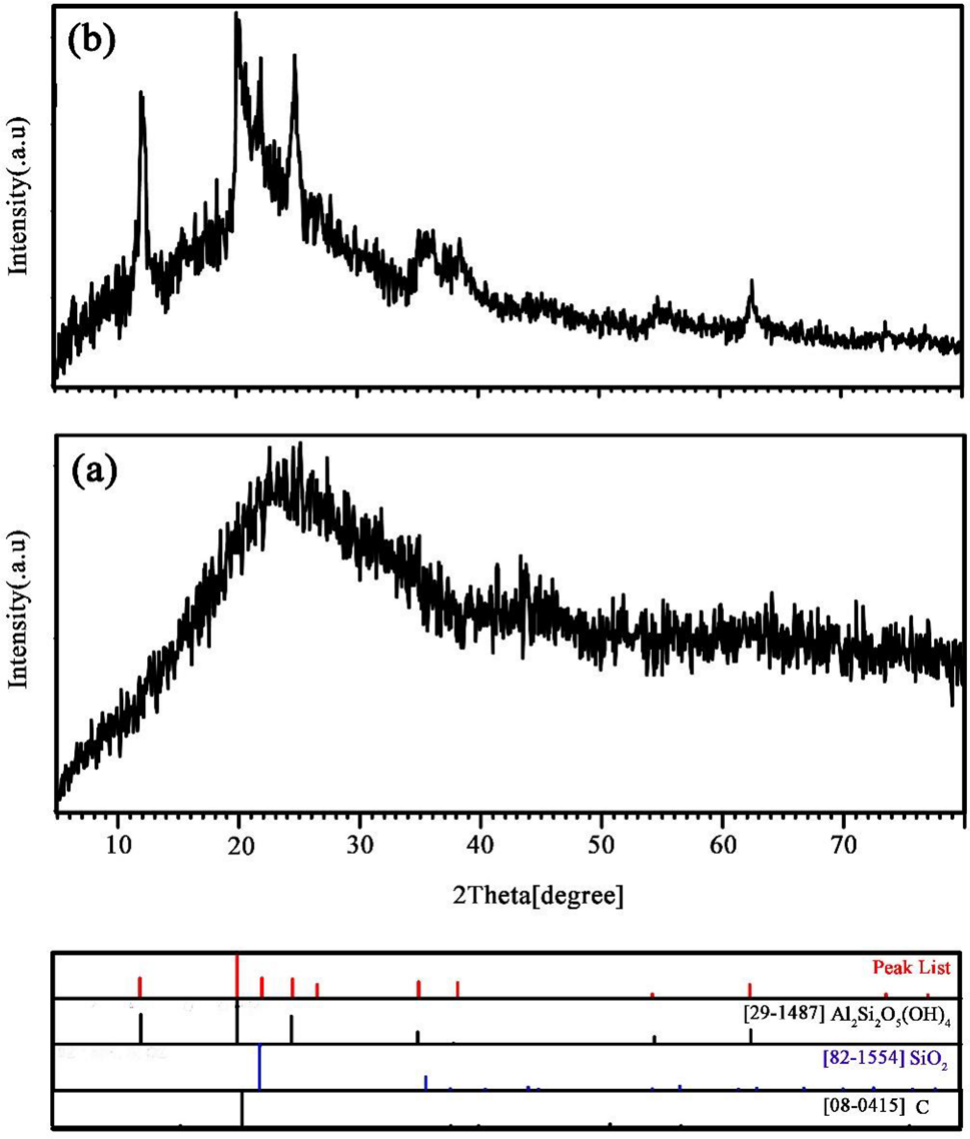
XRD diffractograms of (**a**) pristine S, N-doped GQDs and (**b**) S, N-GQD/HNT nanocomposites.

**Figure 3:**
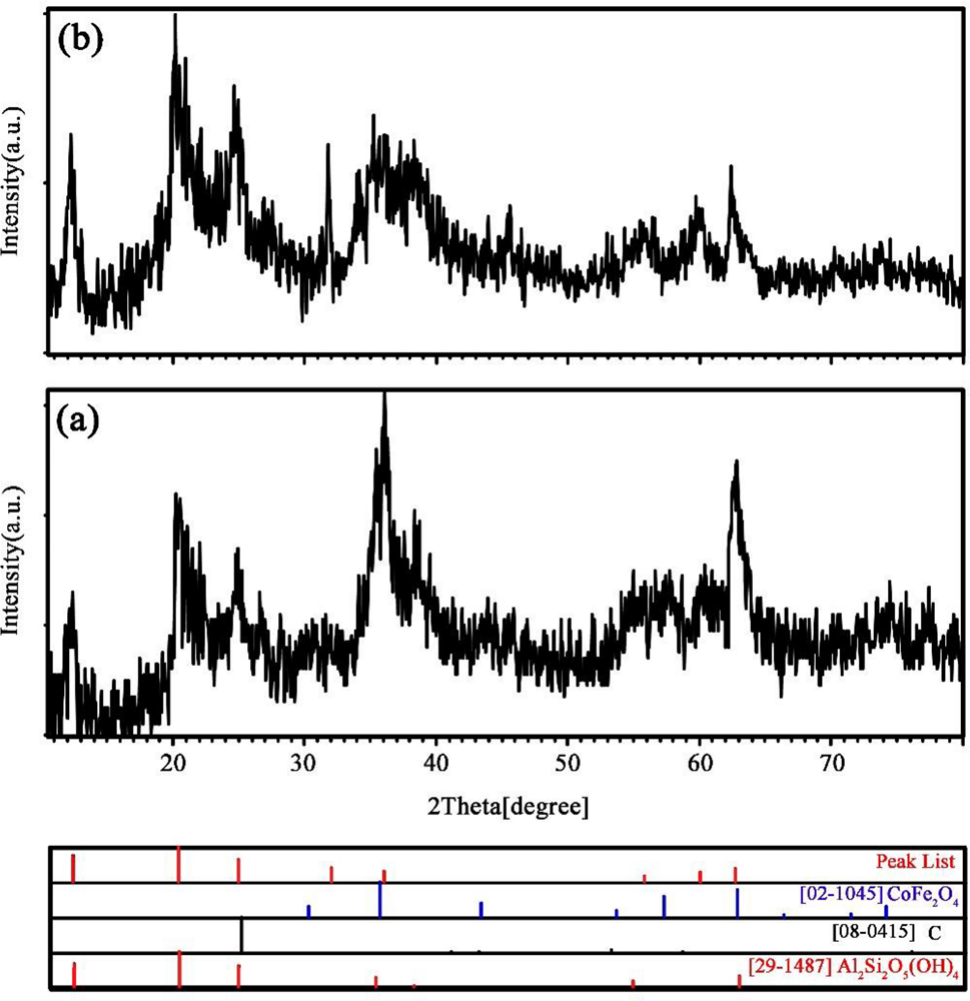
XRD diffractograms of (**a**) CoFe_2_O_4_/HNTs and (**b**) CoFe_2_O_4_/S, N-GQD/HNT nanocomposites.

The halloysite nanotubes were investigated in terms of morphology and purity using FE-SEM micrographs and XRD diffractograms. The schematic diagram for decoration process of S, N-GQDs and CoFe_2_O_4_ nanoparticles on halloysite nanotubes is presented in Fig. 1a–c. Also, the mechanism for hydrogen storage process through spillover represented in Fig. 1d and e. As shown in Fig. S1a and b, the structure of halloysite is nanotubes with different lengths^34^. Additionally, the XRD diffractogram for pristine HNTs is shown in Fig. S1c, which confirms the structure of Al_2_Si_2_O_5_ (OH)_4_ with a reference code of 29–1487. The peak at 2Ɵ = 25 degrees is present in the texture of halloysite nanotube minerals related to SiO_2_ (JCPDS = 82-1554), which is available in clay materials^35,36^. The purity of the as-fabricated pure S, N-GQDs and S, N-GQDs/HNTs nanocomposite samples was studied and is shown in Fig. 2a and b using XRD technique. Figure 2a displays a broad peak at approximately 15–25 degrees of carbon texture, which supports the formation of graphene quantum dots from red onion juice in the hydrothermal process. In addition, the formation of S, N-GQDs on the halloysite nanotube substrate was confirmed by investigation of the XRD diffractogram in Fig. 2b. Figure 2b simultaneous displays the sharp peaks of aluminum silicate hydroxide (29-1487) and silica (82-1554) and a broad peak of carbon (08-0415). In addition, Fig. 3a and b illustrates the XRD diffractograms for CoFe_2_O_4_/HNTs and CoFe_2_O_4_/S, N-GQDs/HNTs, respectively. The presence of cobalt ferrite with a crystal system of cubic (JCPDS = 02-1045) and halloysite nanotubes is available in these two XRD patterns.

**Figure 4:**
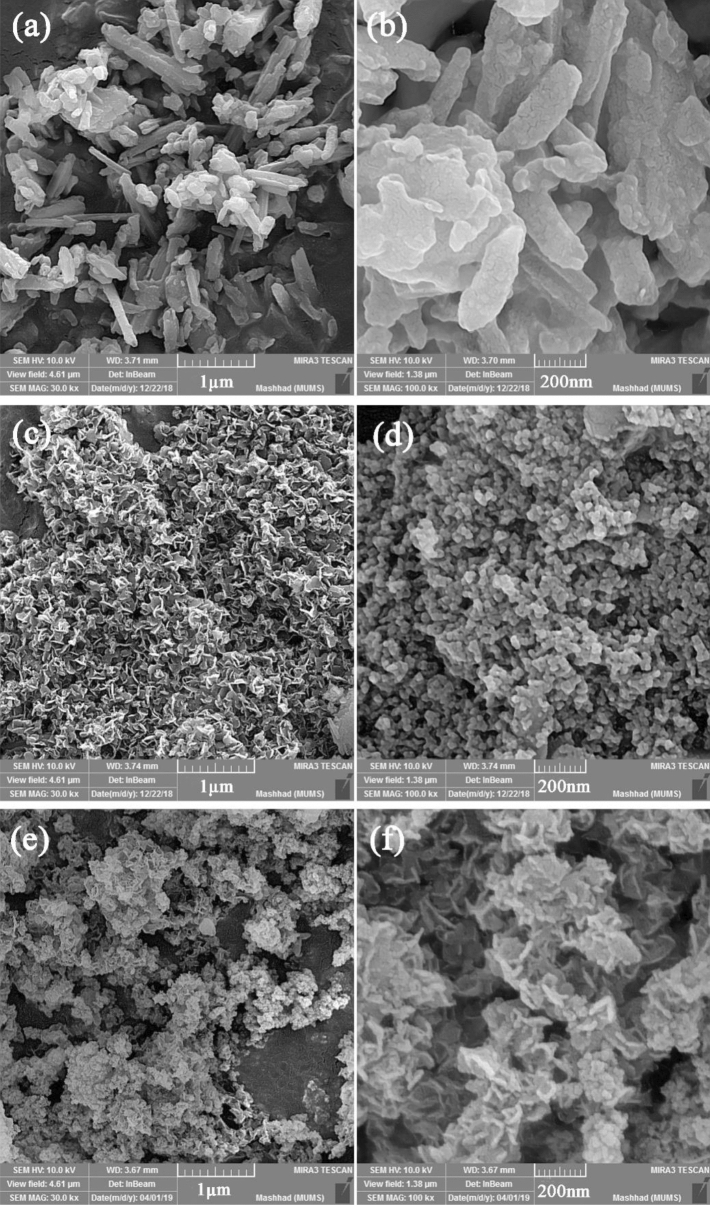
FE-SEM micrographs of (**a**, **b**) S, N-GQDs/HNTs, (**c**, **d**) CoFe_2_O_4_/HNTs and (**e**, **f**) CoFe_2_O_4_/S, N-GQDs/HNTs nanocomposites.

**Figure 5:**
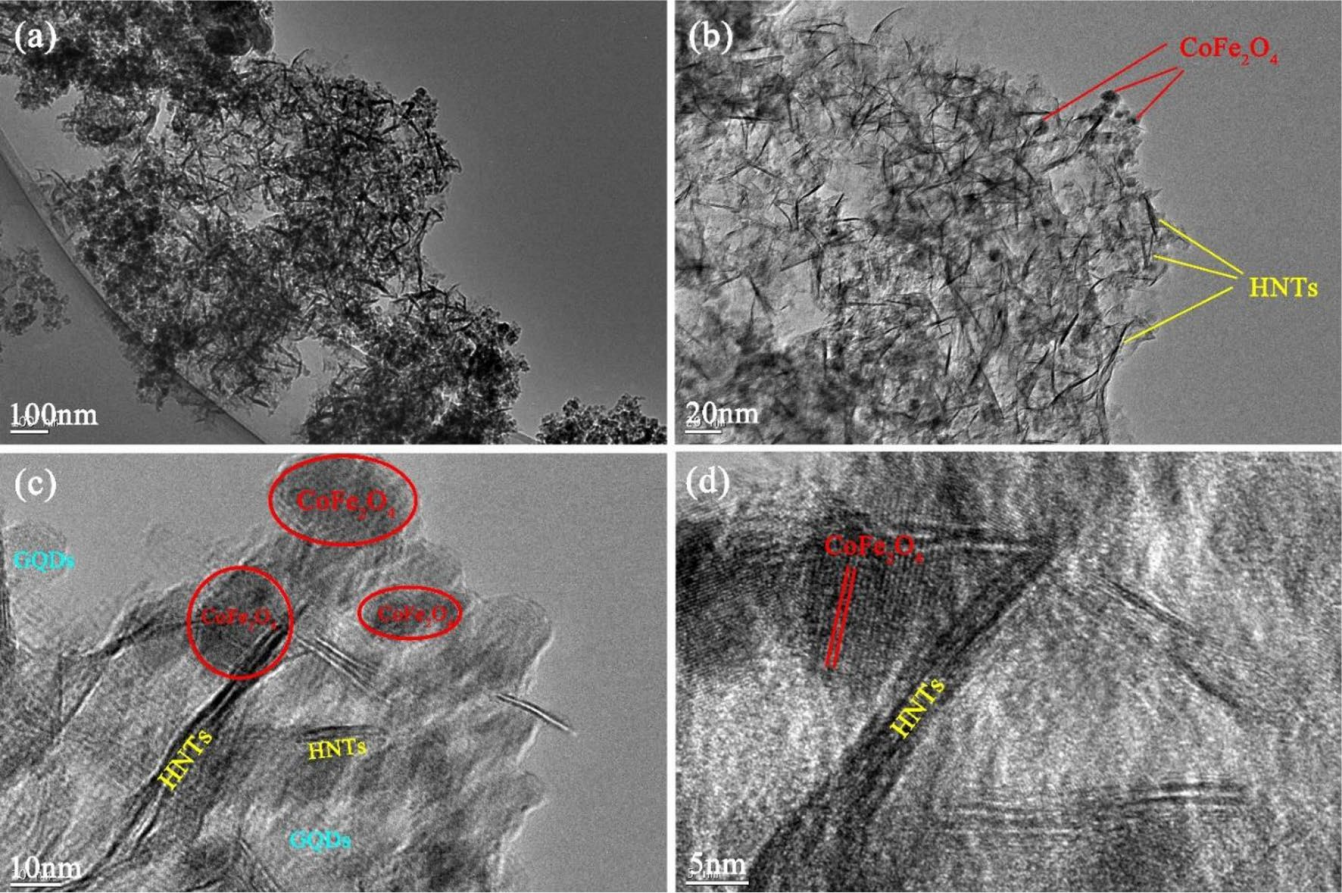
(**a**–**d**) TEM images of CoFe_2_O_4_/S, N-GQD/HNT nanocomposites at different magnifications.

The morphologies of the designed samples, such as S, N-GQDs/HNTs, CoFe_2_O_4_/HNTs and CoFe_2_O_4_/S, N-GQDs/HNTs nanocomposites, are shown in Fig. 4 in different magnifications of 1 µm and 200 nm. Figure 4a and b shows the cover of GQDs on the nanotube of Halloysite clay. The formation of CoFe_2_O_4_ nanoparticles on the HNT structures is displayed in Fig. 4c and d and the creation of CoFe_2_O_4_ and S, N-GQDs on the HNT substrate are displayed in Fig. 4e and f, which leads to the formation of a string of particles on the HNT substrate. Figure 5a–d displays TEM images of designed ternary nanocomposites at various magnifications. The images clearly show the decoration of CoFe_2_O_4_ nanoparticles and S, N-GQDs on the halloysite nanotube substrate. All three components of HNTs, S, N-GQDs and CoFe_2_O_4_ structures are specified in the TEM images. The nanotubes related to halloysite structures and observed particles refer to CoFe_2_O_4_ nanostructures. The measured d-spacing of 0.332 nm correlated with the lattice fringes of CoFe_2_O_4_ is indexed to the lattice spacing of (220) given in Fig. 5d. To better explain the decoration process of S, N-GQDs and CoFe_2_O_4_ nanoparticles on halloysite nanotubes, a schematic diagram is drawn and presented in Fig. 1a–c. This schematic clarified the step-by-step synthesis function in detail.

**Figure 6:**
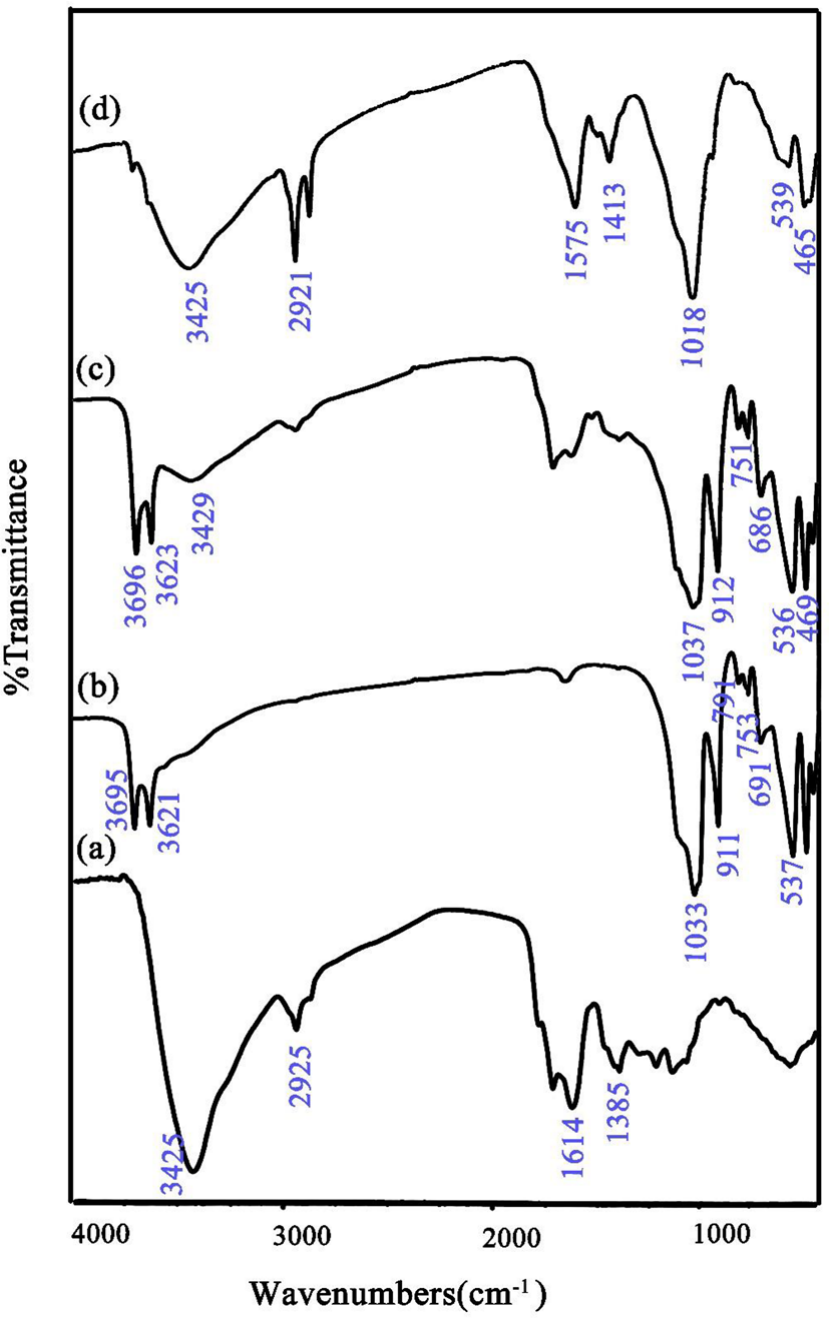
FT-IR spectra of (a) pristine S, N-GQDs, (b) pristine HNTs, (c) S, N-GQDs/HNTs and (d) CoFe_2_O_4_/S, N-GQDs/HNTs nanocomposites.

FT-IR analysis was applied for the detection of chemical bands of samples and is shown in Fig. 6a–d. Figure 6a–d shows the FT-IR spectra of pristine pure S, N-GQDs, pure HNTs, N, S-GQDs/HNTs and CoFe_2_O_4_/S, N-GQDs/HNTs. As seen in the FT-IR spectrum of HNT clay, stretching and bending bonds of Al_2_OH were located at 3695, 3621 and 911 cm^−1^, respectively. The obtained peaks at 753, 691 and 537 cm^−1^ are related to bending bonds of Si–O-Al. The bonds at 1033 cm^−1^ refer to Si–O and OH vibrations in the halloysite units that appear at 791 cm^−1^^37,38^. Metal-oxide vibrations (465 and 539 cm^−1^) in cobalt ferrite and halloysite overlap with each other. The resulting peaks at 1385, 1614 and 2925 cm^−1^ represent the functional groups of GQDs, such as =C–H, C–N, C=O, C–H and C–OH^39^. Elemental analysis of pristine S, N-GQDs and nanocomposites of CoFe_2_O_4_/S, N-GQDs/HNTs is shown in Fig. S2a and b using EDS technique. The presence of sulfur and nitrogen in the EDS analysis of GQDs confirms the formation of S- and N-doped graphene quantum dots. In addition to S, N, and C, the presence of Al, Si, Co, Fe and O affirms the texture of HNTs and CoFe_2_O_4_ in the ternary nanocomposites.

Chemical composition of onion includes several carboxylic acids, sugars, etc. Seven organic acids were identified and quantified in onion samples such as Malic acid, Citric acid, Tartaric acid, Oxalic acid, Ascorbic acid, Succinic acid and Pyruvic acid. Also, sulfur and nitrogen containing compounds detected in the onion extract as organosulfur and amino acids. These compounds consist of 1-Propanethiol, Propylene sulfide, Dimethyl sulfide, Aspartic acid, Serin and, etc.^40,41^.

The possible mechanism of formation of GQDs from the organic acids was explained as follows; the organic acids such as tartaric acid or ascorbic acids when heated at their melting temperature decomposes and the hydronium ion formed from the acid, acted as a catalyst in subsequent decomposition reaction stages. The significant path in mechanism was that the condensation and cyclo-addition followed by the formation of aromatization and aromatic clusters. During the pyrolysis, adjacent dehydrated acid molecules reacted with each other to form GQDs and the functional groups located at the edge of each GQDs acts as passivation layer at the surface^42^. Additionally, presence of sulfur or nitrogen source along with carbon sources leads to formation of S, N doped GQDs. The compounds of thiamine or pantothenic acid in the onion extract appear to be responsible for doping the S or N in the GQDs structures^43^.

The surface specifications were determined by the BET method to compare the surface data of pristine HNTs and representative nanocomposites of CoFe_2_O_4_/S, N-GQDs/HNTs. Surface characterization in terms of specific surface area and porosity is an essential factor for ion adsorption and insertion on the surface or in pores. Therefore, the resulting data can act as important properties for energy storage materials. As seen in Fig. S3a and c, a typical type-IV isotherm can be determined for two BET plots. The obtained isotherms have H3 hysteresis loop. The type-IV isotherm is defined for the materials by mesoporous structures^44^. The specific surface area, total pore volume and average pore diameter for pristine HNTs and nanocomposites of CoFe_2_O_4_/S, N-GQDs/HNTs are summarized in Table S1. The pore size distribution for the samples is shown in Fig. S3b and d. The pore size distribution for pristine HNTs showing broad pore size distribution in the range of 1–55 nm with maximum around pores of 2 nm diameter (Fig. S3b). As shown in Fig. S3d, the pore size distribution for CoFe_2_O_4_/S, N-GQDs/HNTs nanocomposites in the range of 1–45 nm presents the maximum around pores of 40 nm. According to the specific surface area data, by decorating CoFe_2_O_4_ nanoparticles and S, N-GQDs on Halloysite clay, the surface of Halloysite decreases due to covering the surface of HNTs by CoFe_2_O_4_ and S, N-GQD components.

### Electrochemical details

#### Hydrogen storage

**Figure 7:**
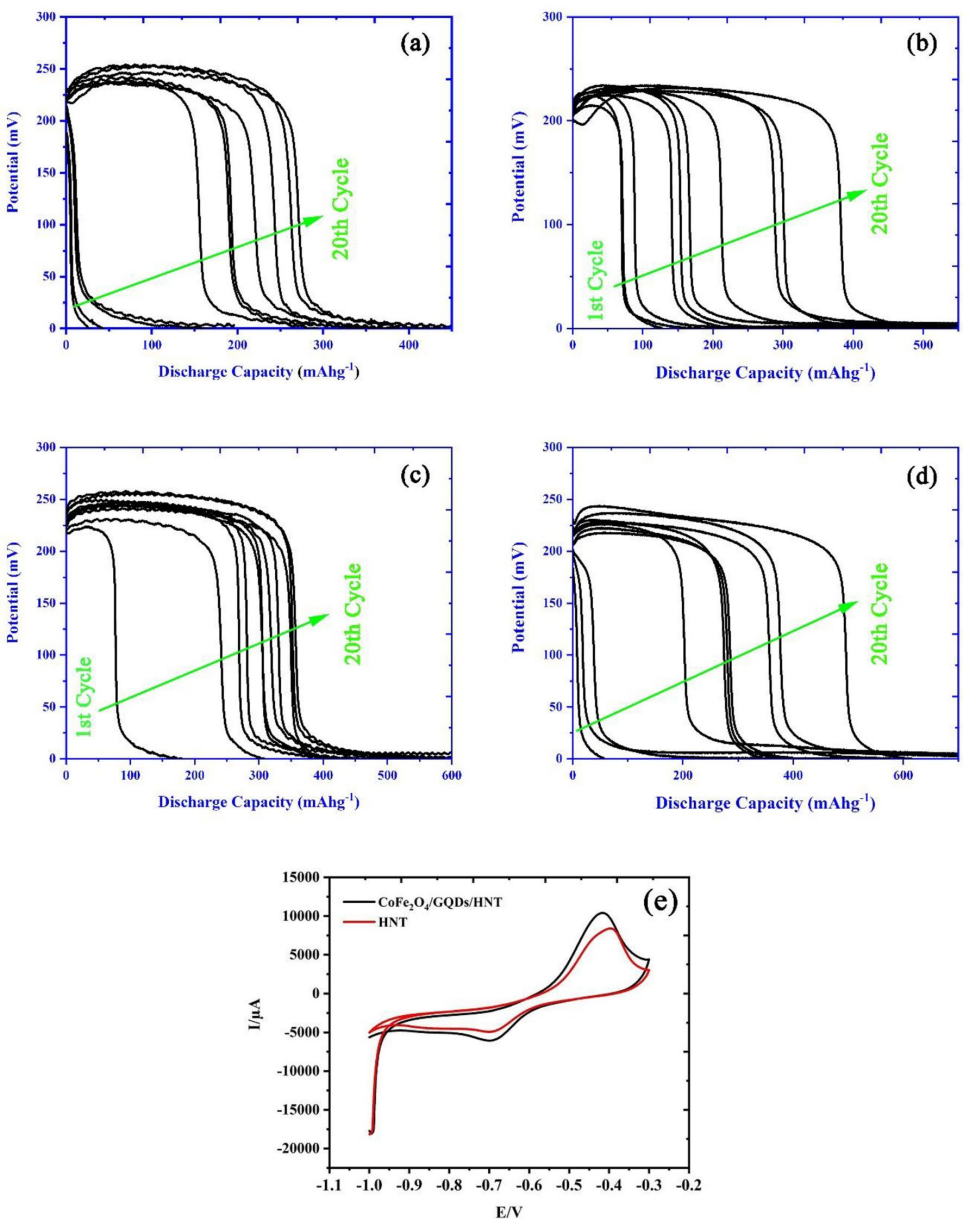
Discharge profiles of (**a**) pristine HNTs, (**b**) CoFe_2_O_4_/HNTs, (**c**) S, N-GQDs/HNTs, (**d**) CoFe_2_O_4_/S, N-GQDs/HNTs nanocomposites and (**e**) comparative cyclic voltammetry (CV) curve for pristine HNTs and CoFe_2_O_4_/S, N-GQDs/HNTs nanocomposites.

Electrochemical hydrogen storage (EHS) capacity is an appropriate criterion for comparing the behavior of active materials in the working electrode for the sorption of hydrogen through the charge–discharge chronopotentiometry (CP) technique. To check this criterion, the designed active materials on the substrate of HNTs were examined in terms of hydrogen storage capacity against pristine HNTs. The discharge profiles for the designed samples of CoFe_2_O_4_/HNTs, S, N-GQDs/HNTs and CoFe_2_O_4_/S, N-GQDs/HNTs are presented in Fig. 7b–d, respectively. As seen in Fig. 7a, the discharge profile for pristine HNTs expresses a discharge capacity of 300 mAhg^-1^ after 20 cycles at a current density of 1 mA. The presence of CoFe_2_O_4_ on the surface of halloysite nanotubes can increase the capacity of HNTs to 450 mAhg^−1^ due to the electrocatalytic task of CoFe_2_O_4_ in the spillover mechanism of clay nanotubes. Additionally, the S, N-GQD/HNT composites show a discharge capacity of 466 mAhg^−1^ after 20 cycles. The simultaneous role of CoFe_2_O_4_ and S, N-GQDs on the surface of HNTs can increase the hydrogen storage capacity of HNTs to 600 mAhg^−1^ by applying a 1 mA current density in a 2 M KOH electrolyte.

The role of hydrogen spillover in hydrogen storage capacity enhancement is still disputable due to lack of understanding of the exact mechanism, and the contrary results on the hydrogen uptake characteristics. Spillover is the dissociation of the hydrogen molecules into atoms by transition metals and subsequent diffusion of these atoms to the host material, in which hydrogen atoms can hydrogenate the unsaturated C–C bonds (i.e., activated carbon, graphite, and single walled carbon nanotubes) or the benzene ring (i.e., MOFs, COFs, and polymers). Among the various steps of spillover, dissociation of hydrogen molecules and diffusion of hydrogen atoms are considered as instantaneous and barrierless, respectively; however, hydrogen atoms need to overcome high energy barriers for migration to the host material which makes migration the rate limiting step. Therefore, minimum clustering and fine molecular level dispersion of transition metals within the host material is desirable to make the migration path as short as possible^45^. Spillover is a conceivable mechanism in hydrogen sorption systems that comprise electrocatalysts to dissociate H_2_ to H atoms. In the spillover concept, adsorbed hydrogen on the electrocatalyst migrates to the surface of support materials such as carbon or layered structures such as graphite or clay, and hydrogen can transport on a surface to another surface. Spillover processes occur in the CoFe_2_O_4_/HNT and CoFe_2_O_4_/S, N-GQD/HNT composites when hydrogen is adsorbed on the CoFe_2_O_4_ nanoelectrocatalysts and two-step spillover occurs. Primary spillover occurs through transportation of atomic hydrogen from the electrocatalyst nanoparticle to the support, and secondary spillover occurs through transportation of hydrogen to the receptor (HNT). The diffusion procedure is obligatory for secondary spillover^46^. Therefore, the formation of carbon bridges (S, N-GQDs) between the dissociation source (CoFe_2_O_4_) and receptor (HNTs) leads to diffusion. Secondary spillover was substantiated to develop the hydrogen storage capacity by mixing the adsorbent with a catalyst that is capable of dissociating hydrogen^47,48^. The explained spillover mechanism in the CoFe_2_O_4_/S, N-GQDs/HNTs nanocomposites is shown in detail in Fig. 1d and e.

In addition to the role of the carbon bridge of S, N-GQDs in the spillover mechanism, the hydrogen sorption ability in the presence of codoped doped GQDs (CoFe_2_O_4_/S, N-GQDs/HNTs) is better than that of CoFe_2_O_4_/HNTs owing to the electron transfer in the S, N-GQD-based electrodes being properly quick. The formation of S, N-GQDs on electrodes can alter the discharge capacity because of the numerous edge areas produced by small sized S, N-GQDs and the adequate conductivity correlated to CoFe_2_O_4_ bond structures. The high efficiency of CoFe_2_O_4_/S, N-GQDs/HNTs can be elucidated owing to the trap states formed by both dopants and edge states, which can adsorb charge carriers to increase the storage capability. The electron density of graphene layers can be modified through doping by electron acceptor or electron donor species. Sulfur or nitrogen as heteroatoms in the graphene structure can be replaced with carbon atoms. Therefore, electron migration occurs, which leads to the creation of electrons and holes. This structure alters with cooperating S, N-GQDs and plays a great role in electrochemical and electrocatalyst systems for energy storage applications^22,49^. Additionally, nitrogen as a donor atom with the ability to electronically modify graphene can improve the conductivity and catalysis behaviors of graphene structures^50,51^.

Operation of hydrogen sorption in metal oxides situates through physisorption. The produced H^+^ from aqueous electrolyte adsorbs on the surface of the metal oxide (charge process). During the discharge path, H_2_ migrates from the working electrode under alkaline circumstances and becomes water again while freeing an electron. In the next step, adsorbed hydrogen on the electrocatalyst of metal oxides migrates from nanoparticles to the surface of the carbon support, and hydrogen can be transported on a surface to another surface^52^.

Actually, the Heyrovsky process occurred due to the suitable electrocatalytic activity of synthesized CoFe_2_O_4_ nanoparticles^53^. The synergic effect of Tafel [Eq. (2)]^54^ and Heyrovsky [Eq. (3)] process leads to hydrogen storage in the active materials.

1 $$ {\text{CoFe}}_{{2}} {\text{O}}_{{4}} /{\text{S}},{\text{ N}} - {\text{GQDs}}/{\text{HNTs }} + {\text{ H}}_{{2}} {\text{O }} + {\text{ e}}^{ - } \leftrightarrow {\text{ CoFe}}_{{2}} {\text{O}}_{{4}} /{\text{S}},{\text{ N}} - {\text{GQDs}}/{\text{HNTs }} - {\text{H}}_{{{\text{ads}}}} + {\text{ OH}}^{ - } $$

2 $$ {\text{H }} + {\text{ H }} \leftrightarrow {\text{ H}}_{{2}} $$

3 $$ {\text{CoFe}}_{{2}} {\text{O}}_{{4}} /{\text{S}},{\text{ N}} - {\text{GQDs}}/{\text{HNTs }} - {\text{H}}_{{{\text{ads}}}} +_{{}} {\text{CoFe}}_{{2}} {\text{O}}_{{4}} /{\text{S}},{\text{ N}} - {\text{GQDs}}/{\text{HNTs }} - {\text{H}}_{{{\text{ads}}}} \leftrightarrow {\text{ 2 CoFe}}_{{2}} {\text{O}}_{{4}} /{\text{S}},{\text{ N}} - {\text{GQDs}}/{\text{HNTs }} + {\text{H}}_{{2}} $$

4 $$ {\text{H}}_{{2}} {\text{O }} + {\text{ e}}^{ - } \leftrightarrow {\text{ H }} + {\text{ OH}}^{ - } $$

5 $$ {\text{CoFe}}^{{({\text{III}})}}_{{2}} {\text{O}}_{{4}} + {\text{ XH}}_{{2}} {\text{O }} + {\text{ xe}}^{ - } \leftrightarrow {\text{ CoFe}}^{{({\text{III}})}}_{{{2} - {\text{x}}}} {\text{Fe}}^{{({\text{II}})}}_{{\text{x}}} {\text{H}}^{{({\text{I}})}}_{{\text{x}}} {\text{O}}_{{4}} + {\text{ xOH}}^{ - } $$

According to Eq. (4), the electrolyte of KOH dissociated and the ions of OH^−^ and H^+^ were produced during the charge direction. Next, the produced H^+^ migrates to the working electrode. In the cathodic reaction, the H^+^ adsorbs on the surface of active materials. During an oxidization process and anodic reaction, the charging mechanism was performed in the counter electrodes (Pt). During the discharge direction, the stored H_2_ in the working electrode migrates through freeing an electron under alkaline circumstances and water is again produced. The Fe^3+^ as a central metal ion is reduced to Fe^2+^ [Eq. (5)]. Finally, the adsorbing H^+^ can equilibrate the total charge of CoFe_2_O_4_. The Fe-H bonds created in terms of construction favored the formation of OH^−^ ions with a reduction in the redox couple of Fe^3+^/Fe^2+^.

#### Cyclic voltammetry

The electroanalytical technique of cyclic voltammetry (CV) was customarily employed for the comparative study of electrode material attributes. Fascinating and effective scientific insights can be provided by associating the attributes of the material, such as structure and morphology, with their electrochemical response. The electrochemical response of HNTs decorated with CoFe_2_O_4_ and GQDs was investigated by CV for comparison with pristine HNTs. Figure 7e exhibits the CV curves for the pristine HNTs and representative nanocomposites of CoFe_2_O_4_/S, N-GQDs/HNTs over a potential region of − 1.1 to − 0.2 V. The electrode response (anodic and cathodic peaks) for all samples is listed in Table S2. The CoFe_2_O_4_/S, N-GQDs/HNT nanocomposites express a better electrode response than pristine HNTs. It is fascinating to note that covering S, N-GQDs on HNT structures modifies the electrochemical attributes of the as-fabricated electrode materials for energy storage applications. Additionally, CoFe_2_O_4_ as an electrocatalyst can improve the energy storage mechanism, which is compatible with chronopotentiometry charge–discharge results.

## Conclusion

Halloysite nanoclay as a unique substrate shows favorable properties in terms of hydrogen adsorption using incorporating physisorption and spillover mechanisms. The green synthesized Sulfur and Nitrogen co-doped graphene quantum dots as conductive components and cobalt ferrite nanoparticles as electrocatalyst was decorated on the halloysite substrate. The morphology and purity of designed ternary nanocomposites confirm the presence of CoFe_2_O_4_ and S, N-GQDs on the HNTs substrate. The halloysite and treated halloysite show hydrogen storage capacity of 300 and 600 mAhg^−1^ compared to each other in the 2 M KOH electrolyte after 20 cycles at a current of 1 mA through the charge/discharge chronopotentiometry method. As a result, the fabricated CoFe_2_O_4_/S, N-GQS/HNTs nanocomposites provide a facile process for the reflective plan of potential compounds for energy storage devices.

## Methods

### Precursors and materials

The chemical precursors and starting materials utilized in the synthesis process of samples, including halloysite nanotubes (HNTs), FeCl_3_·6H_2_O, Co(CH_3_CO_2_)_2_·4H_2_O, CTAB and NaOH, were purchased from a Merck company and applied without further purification. XRD diffractograms were recorded by an X-ray diffractometer device using Ni-filtered Cu Ka radiation (Philips-X’pertpro). FT-IR spectra were obtained on a Nicolet Magna-550 spectrometer in KBr pellets. SEM micrographs were obtained on a LEO-1455VP equipped with energy dispersive X-ray spectroscopy. EDS analysis with a 20 kV hasten voltage was performed. TEM images were captured on a Philips EM208 transmission electron microscope with an accelerating voltage of 200 kV. The BET analysis was conducted at − 196 °C using an automated gas adsorption analyzer (Tristar 3000, Micromeritics). The distribution of pore size was measured by applying the desorption branch of the isotherm in the BJH method.

### Green synthesis of S, N-GQDs

The red onion was selected as the green source for the synthesis of graphene quantum dots using a hydrothermal procedure. To use onion extract, the fresh red onion was squeezed, and the onion juice was filtered from the pulps. The red onion juice was filtered again and transferred to a 50 ml sealed autoclave and heated for 8 h at 180 °C. The obtained product was filtered, and the GQD solution was separated from the resulting powders. The obtained solution contains an S, N-codoped GQD solution.

### Preparation of S, N-GQDs/HNTs

To synthesize S, N-GQDs on the substrate of halloysite nanotubes, 0.2 g HNTs were dispersed in 50 ml diluted red onion juice using an ultrasonic bath. The prepared mixture was transferred to a sealed autoclave and heated for 8 h at 180 °C. Finally, the resulting products of S, N-GQDs/HNTs were dried for 24 h at 80 °C in a vacuum oven.

### Preparation of CoFe_2_O_4_/S, N-GQDs/HNTs

The S, N-GQD/HNT powders provided in “Electrochemical details” were used as substrates for the synthesis of CoFe_2_O_4_ nanoparticles. S (0.02 g), N-GQD/HNT powders ultrasonically dispersed in DI water. Then, the mixed solution comprises 0.13 g FeCl_3_·6H_2_O, 0.10 g Co(CH_3_CO_2_)_2_·4H_2_O and 0.13 g CTAB in DI water prepared and added to the dispersed solution of S, N-GQDs/HNTs. The pH of the provided mixture was adjusted to 11 using NaOH and stirred for 20 min. Finally, the resulting mixture was transferred to a sealed autoclave and heated for 15 h at 150 °C. The prepared CoFe_2_O_4_ on the substrate of S, N-GQDs/HNTs were centrifuged and dried at 60 °C in an oven.

### Electrochemical experiments

The collected cells possessed the resulting electrode material for the working electrode, platinum plate as a counter electrode, Ag/AgCl as a reference electrode and KOH in DI water as the electrolyte. The working electrode was designed by coating the synthesized materials on the copper substrate. In three-electrode cells, it is necessary to calculate the potential changes of the working electrode, so this change is studied with respect to a reference electrode (a potentially defined electrode). These electrodes provide the facility of specifying the working electrode potential by providing constant and repeatable potential. Ag/AgCl electrodes are the most widely used reference electrodes in this system^55^. These resulting copper substrates were applied as working electrodes in three-electrode hydrogen storage cells. Galvanostatic charge–discharge investigations were performed on the SAMA 500 testing device at desired current densities. Cyclic voltammogram experiments for the obtained electrodes were performed in similarly collected cells at a scan rate of 0.10 Vs^−1^.

## Supplementary Information

Supplementary Information.
