# Author Correction: Decoration of green synthesized S, N-GQDs and CoFe_2_O_4_ on halloysite nanoclay as natural substrate for electrochemical hydrogen storage application

**DOI:** 10.1038/s41598-022-17519-y

**Published:** 2022-08-02

**Authors:** Maryam Ghiyasiyan-Arani, Masoud Salavati-Niasari

**Affiliations:** Institute of Nano Science and Nano Technology, University of Kashan, P. O. Box. 87317-51167, Kashan, Islamic Republic of Iran

Correction to: *Scientific Reports* 10.1038/s41598-022-12321-2, published online 16 May 2022

In the original version of this Article, Masoud Salavati-Niasari was omitted as a corresponding author. Correspondence and requests for materials should also be addressed to Salavati@kashanu.ac.ir.

In addition, the original version of this Article contained an error in the Acknowledgments section.

“Professor Masoud Salavati-Niasari and coauthors of the Institute of Nano Science and Nano Technology, University of Kashan, IRAN, are kindly acknowledged for providing access to the analysis facility by Grant No. 97014327 from University of Kashan and the council of Iran National Science Foundation (INSF) by Grant No. 99019327.”

now reads:

“Professor Masoud Salavati-Niasari and coauthors of the Institute of Nano Science and Nano Technology, University of Kashan, IRAN, are kindly acknowledged for providing access to the analysis facility by Grant No. (159271/MG8) from University of Kashan and the council of Iran National Science Foundation (INSF) by Grant No. (97017837).”

The original Article has been corrected.

