# Supplementary material for: Decoration of green synthesized S, N-GQDs and CoFe2O4 on halloysite nanoclay as natural substrate for electrochemical hydrogen storage application
Source: Sci Rep. 2022 May 16;12:8103. doi: 10.1038/s41598-022-12321-2 (PMC9110390; doi:10.1038/s41598-022-12321-2)
Supplement: Supplementary file 1 — Supplementary Information. [file 41598_2022_12321_MOESM1_ESM.docx]

**Supporting Information**

**Decoration of Green Synthesized S, N-GQDs and CoFe_2_O_4_ on Halloysite Nanoclay as Natural Substrate for Electrochemical Hydrogen Storage Application**

**Maryam Ghiyasiyan-Arani*, Masoud Salavati-Niasari**

*Institute of Nano Science and Nano Technology, University of Kashan, Kashan, P. O. Box.87317-51167, I. R. Iran*

** Corresponding author. Tel.: +98 315 5912383; Fax: +98 315 5552935; E-mail address:* m.ghiyasiyan@grad.kashanu.ac.ir


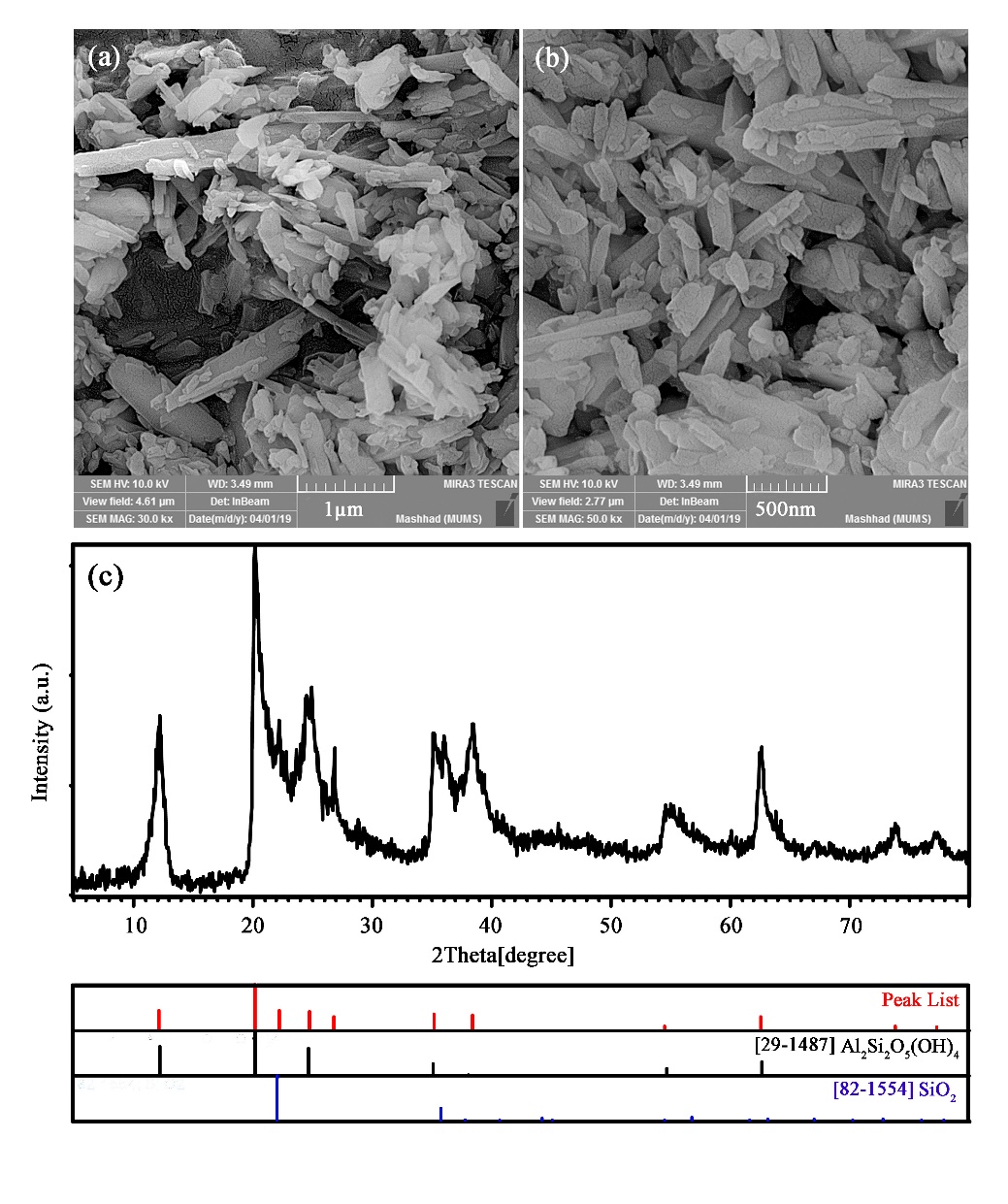


**Fig. S1:** (a, b) FE-SEM micrographs and (c) XRD diffractogram of pristine HNTs


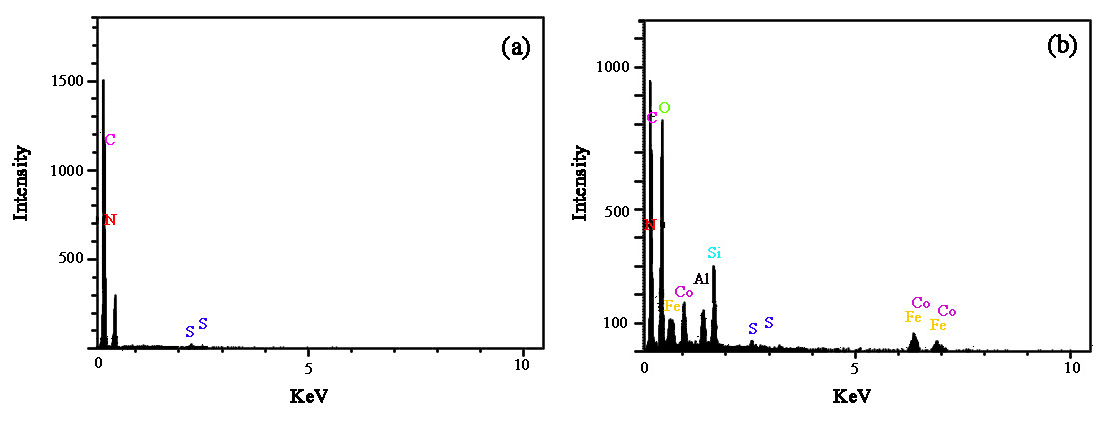


**Fig. S2:** EDS analysis of (a) pristine S, N-GQDs and (b) CoFe_2_O_4_/S, N-GQDs/ HNTs nanocomposites.


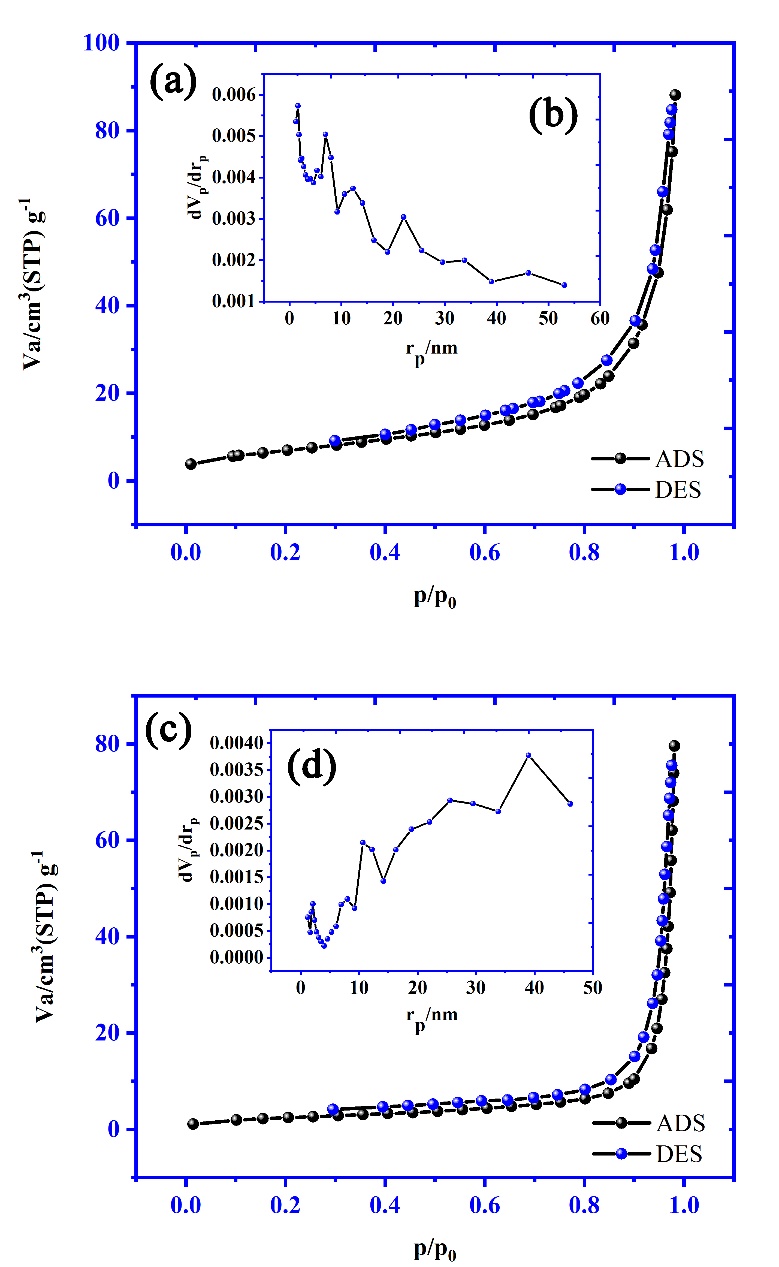


**Fig. S3:** BET and BJH results of (a, b) pristine HNTs and (c, d) nanocomposites of CoFe_2_O_4_/S,N-GQDs/HNTs

**Table.S1:** Summarized BET-BJH specifications Data

| **Sample** | **S_BET_ (m^2^g^-1^)** | **Total pore volume (cm^3^g^-1^)** | **D_avg_ (nm)** |
| --- | --- | --- | --- |
| **HNTs** | 25.324 | 0.1362 | 21.517 |
| **CoFe_2_O_4_/S, N-GQDs/HNTs** | 8.897 | 0.1231 | 55.346 |

**Table S2:** summarized cyclic voltammetry data for pristine HNTs and CoFe_2_O_4_/S, N-GQDs/HNTs nanocomposites.

|  | **I_Pa_** **(µA)** | **I_Pc_ (µA)** | **E_Pa_ (V)** | **E_Pc_ (V)** |
| --- | --- | --- | --- | --- |
| **HNTs** | 8409 | -4927 | -0.395 | -0.697 |
| **CoFe_2_O_4_/S, N-GQDs/HNTs** | 10409 | -6060 | -0.418 | -0.696 |
